# Hats and Baldness

**Published:** 1853-05

**Authors:** 


### ART. VI. — *Hats and Baldness.*

The following letter has been received since our last issue. We heartily sympathize with our correspondent, and regret, on his account, as well as in behalf of many others, that the promulgation of our discovery comes too late for their benefit. They must comfort themselves by the reflection that if the needed reform be accomplished, a valuable boon will be conferred on future generations!

ALMOND, *March* 21, 1853.

SIR,—Having just seen your article on baldness in the Journal, and being unfortunately one of the many to whom it addresses itself with peculiar force, I acknowledge the justice of your remark, that any allusion to one's bald pate "leaves a sadness at the bottom," as it reminds him of the loss of that appendage of manly beauty, and strength, of which Delilah bereft Sampson, the loss of which was followed by such sad consequences to the Jewish champion.

I well remember the first symptoms of baldness which I felt. Before I became aware that my hair was falling—a soreness on the crown of my head, in the morning when brushing my hair—and had I then been aware of the cause, I might not now be obliged to deplore its loss. I am now forty-four years of age, generally well, hale and hearty, and though time has laid his hand on me but slightly in other respects, yet this depilous condition of my vertex, and certain crow-foot appearances visible in the corner of my eyes, admonish me, as I in vain attempt to cover the denuded portion of my caput from the temporal regions,—thank God! I am not gray—that I must soon pass off to make room for those who are to come after. With my present experience, aided by your views, I would eschew hats forever, and wear turbans "a la Turk," or anything else rather than lose my fair locks.

"What in the whole work of art," says a late writer, "is more unnatural, stiff and uncomfortable than a modern hat—a mass of glue, paper, and wool, formed into a cone or circle, with hard lines and stiff unyielding corners presented to view on all sides, without beauty, or grace, or comfort, or even use," &c., &c.

As the ladies *have donned* some portions of the male attire, I would suggest the propriety of gentlemen adopting the head-dress of the fair sex, which it appears is so efficacious in preventing baldness.

I go for a change—for the bonnet—perhaps; altered a little, so as to give it more of the air of the male costume—if for nothing else than to *be revenged* on the ladies—*provoking creatures*—for adopting the pants. It might be so constructed as to appear graceful, to fit easily, to set lightly on the head, and be surmounted by a plume, after the fashion of the Kossuth. But cannot something *be done* to do away the tyranny of fashion in this article of our attire, which is not only worthless for comfort or beauty, but positively injurious, as whatever interrupts the circulation in the scalp, may, through the vascular connection existing between it and the brain, induce disease of that organ? A SUBSCRIBER.

